# Supplementary material for: The C-terminal tail of CSNAP attenuates the CSN complex
Source: Life Sci Alliance. 2023 Jul 17;6(10):e202201634. doi: 10.26508/lsa.202201634 (PMC10355216; doi:10.26508/lsa.202201634)
Supplement: Supplementary file 1 [file LSA-2022-01634_TableS1.docx]

| **Table 1 – Peptide array data for rCSN^WT^ used in Fig.3.** | | | | **intensities as fold of C-CSNAP (A1)** | | | | | |  |  |
| --- | --- | --- | --- | --- | --- | --- | --- | --- | --- | --- | --- |
|  |  |  |  | Experiment #1 | | Experiment #2 | | Experiment #3 | |  |  |
|  | **Residues** | **Position** | **sequence** | **left** | **right** | **left** | **right** | **left** | **right** | **Average fold change** | **standard error** |
| **Elongation** | **42-57** | A 1 | DFFNDFEDLFDDDDIQ | 1.0000 | 1.0000 | 1.0000 | 1.0000 | 1.0000 | 1.0000 | **1.0000** | 0.0000 |
|  | **41-57** | A 2 | ADFFNDFEDLFDDDDIQ | 0.9064 | 0.9697 | 0.9657 | 0.9368 | 0.9189 | 1.1256 | **0.9705** | 0.0267 |
|  | **40-57** | A 3 | HADFFNDFEDLFDDDDIQ | 0.9340 | 0.9213 | 1.0742 | 1.0023 | 0.9272 | 1.3368 | **1.0326** | 0.0534 |
|  | **39-57** | A 4 | VHADFFNDFEDLFDDDDIQ | 0.8499 | 1.0011 | 1.1271 | 1.1074 | 1.0043 | 1.6241 | **1.1190** | 0.0888 |
|  | **38-57** | A 5 | AVHADFFNDFEDLFDDDDIQ | 0.9283 | 0.9891 | 1.1636 | 1.1086 | 1.0248 | 1.6430 | **1.1429** | 0.0863 |
|  | **37-57** | A 6 | KAVHADFFNDFEDLFDDDDIQ | 0.9545 | 0.8998 | 1.1773 | 1.1039 | 0.9022 | 1.5774 | **1.1025** | 0.0862 |
|  | **36-57** | A 7 | EKAVHADFFNDFEDLFDDDDIQ | 1.0047 | 0.9406 | 1.2325 | 1.0510 | 1.0404 | 1.2208 | **1.0817** | 0.0396 |
|  | **35-57** | A 8 | NEKAVHADFFNDFEDLFDDDDIQ | 0.9774 | 0.9368 | 1.1059 | 0.9077 | 1.0678 | 1.5735 | **1.0949** | 0.0822 |
|  | **34-57** | A 9 | ANEKAVHADFFNDFEDLFDDDDIQ | 1.0621 | 0.9059 | 1.0725 | 0.8763 | 1.1445 | 1.4400 | **1.0836** | 0.0676 |
|  | **33-57** | A10 | AANEKAVHADFFNDFEDLFDDDDIQ | 1.0080 | 0.7984 | 1.1744 | 0.8665 | 0.9505 | 1.6976 | **1.0826** | 0.1092 |
|  | **32-57** | A11 | LAANEKAVHADFFNDFEDLFDDDDIQ | 1.0973 | 0.8004 | 1.2354 | 0.8692 | 1.0483 | 1.7633 | **1.1357** | 0.1151 |
|  | **31-57** | A12 | DLAANEKAVHADFFNDFEDLFDDDDIQ | 0.9578 | 0.6616 | 1.1101 | 0.7799 | 0.9781 | 1.4995 | **0.9978** | 0.0974 |
|  | **30-57** | A13 | MDLAANEKAVHADFFNDFEDLFDDDDIQ | 1.1276 | 0.6511 | 1.2073 | 0.7597 | 0.8915 | 1.4663 | **1.0173** | 0.1018 |
|  | **29-57** | A14 | LMDLAANEKAVHADFFNDFEDLFDDDDIQ | 1.0025 | 0.5569 | 1.0973 | 0.7377 | 0.8156 | 1.3473 | **0.9262** | 0.0939 |
|  | **28-57** | A15 | LLMDLAANEKAVHADFFNDFEDLFDDDDIQ | 1.1496 | 0.5625 | 1.1572 | 0.6698 | 0.8606 | 1.4628 | **0.9771** | 0.1133 |
|  | **27-57** | A16 | GLLMDLAANEKAVHADFFNDFEDLFDDDDIQ | 1.2014 | 0.5729 | 1.1811 | 0.6693 | 0.7975 | 1.6779 | **1.0167** | 0.1386 |
|  | **26-57** | A17 | TGLLMDLAANEKAVHADFFNDFEDLFDDDDIQ | 1.1089 | 0.7213 | 1.2861 | 0.6911 | 0.9681 | 1.4680 | **1.0406** | 0.1030 |
|  | **25-57** | A18 | STGLLMDLAANEKAVHADFFNDFEDLFDDDDIQ | 1.0821 | 0.6332 | 1.1536 | 0.6103 | 0.9488 | 1.3319 | **0.9600** | 0.0966 |
|  | **24-57** | A19 | GSTGLLMDLAANEKAVHADFFNDFEDLFDDDDIQ | 1.0061 | 0.4837 | 1.0369 | 0.5457 | 0.7458 | 1.0844 | **0.8171** | 0.0876 |
|  | **23-57** | A20 | GGSTGLLMDLAANEKAVHADFFNDFEDLFDDDDIQ | 1.1397 | 0.5669 | 1.0788 | 0.5519 | 0.7645 | 1.1742 | **0.8793** | 0.0957 |
|  | **22-57** | A21 | AGGSTGLLMDLAANEKAVHADFFNDFEDLFDDDDIQ | 1.2564 | 0.6419 | 1.3584 | 0.6324 | 0.7519 | 1.3089 | **0.9917** | 0.1168 |
|  | **21-57** | A22 | EAGGSTGLLMDLAANEKAVHADFFNDFEDLFDDDDIQ | 1.3757 | 0.8842 | 1.3407 | 0.7915 | 0.7892 | 1.6752 | **1.1428** | 0.1240 |
|  | **20-57** | A23 | DEAGGSTGLLMDLAANEKAVHADFFNDFEDLFDDDDIQ | 1.2835 | 0.8256 | 1.3390 | 0.7175 | 0.7580 | 1.5127 | **1.0727** | 0.1150 |
|  | **19-57** | A24 | LDEAGGSTGLLMDLAANEKAVHADFFNDFEDLFDDDDIQ | 0.7935 | 0.4198 | 0.9139 | 0.5720 | 0.6115 | 0.9977 | **0.7181** | 0.0736 |
|  | **18-57** | B 1 | DLDEAGGSTGLLMDLAANEKAVHADFFNDFEDLFDDDDIQ | 1.0274 | 1.1571 | 1.1352 | 1.0000 | 1.0228 | 1.6798 | **1.1704** | 0.0859 |
|  | **17-57** | B 2 | VDLDEAGGSTGLLMDLAANEKAVHADFFNDFEDLFDDDDIQ | 0.7459 | 0.5916 | 0.7782 | 0.9368 | 0.7157 | 0.9775 | **0.7909** | 0.0480 |
|  | **16-57** | B 3 | YVDLDEAGGSTGLLMDLAANEKAVHADFFNDFEDLFDDDDIQ | 1.0719 | 1.0102 | 1.1786 | 1.0023 | 0.9261 | 1.4758 | **1.1108** | 0.0659 |
|  | **15-57** | B 4 | PYVDLDEAGGSTGLLMDLAANEKAVHADFFNDFEDLFDDDDIQ | 0.8176 | 0.7127 | 0.9836 | 1.1074 | 0.9819 | 1.4893 | **1.0154** | 0.0902 |
|  | **14-57** | B 5 | GPYVDLDEAGGSTGLLMDLAANEKAVHADFFNDFEDLFDDDDIQ | 0.9007 | 1.0247 | 1.1048 | 1.1086 | 0.9314 | 1.5747 | **1.1075** | 0.0815 |
|  | **13-57** | B 6 | AGPYVDLDEAGGSTGLLMDLAANEKAVHADFFNDFEDLFDDDDIQ | 0.7221 | 0.7847 | 1.0724 | 1.1039 | 0.8260 | 1.6222 | **1.0219** | 0.1110 |
|  | **12-57** | B 7 | GAGPYVDLDEAGGSTGLLMDLAANEKAVHADFFNDFEDLFDDDDIQ | 0.8903 | 1.1085 | 1.2397 | 1.0510 | 0.9896 | 1.7387 | **1.1696** | 0.1008 |
|  | **11-57** | B 8 | EGAGPYVDLDEAGGSTGLLMDLAANEKAVHADFFNDFEDLFDDDDIQ | 0.7377 | 0.7027 | 0.9362 | 0.9077 | 0.7646 | 1.2161 | **0.8775** | 0.0636 |
|  | **10-57** | B 9 | PEGAGPYVDLDEAGGSTGLLMDLAANEKAVHADFFNDFEDLFDDDDIQ | 0.9905 | 0.9358 | 1.1600 | 0.8763 | 0.9713 | 1.7258 | **1.1099** | 0.1054 |
|  | **9-57** | B10 | FPEGAGPYVDLDEAGGSTGLLMDLAANEKAVHADFFNDFEDLFDDDDIQ | 0.8089 | 0.7335 | 0.9593 | 0.8665 | 0.8826 | 1.6065 | **0.9762** | 0.1060 |
|  | **8-57** | B11 | MFPEGAGPYVDLDEAGGSTGLLMDLAANEKAVHADFFNDFEDLFDDDDIQ | 0.9572 | 0.8391 | 1.0458 | 0.8692 | 0.8450 | 1.5649 | **1.0202** | 0.0928 |
|  | **7-57** | B12 | EMFPEGAGPYVDLDEAGGSTGLLMDLAANEKAVHADFFNDFEDLFDDDDIQ | 0.7210 | 0.6454 | 0.8666 | 0.7799 | 0.7015 | 1.6281 | **0.8904** | 0.1230 |
|  | **6-57** | B13 | DEMFPEGAGPYVDLDEAGGSTGLLMDLAANEKAVHADFFNDFEDLFDDDDIQ | 0.8904 | 0.8906 | 1.1179 | 0.7597 | 0.8884 | 1.6152 | **1.0270** | 0.1035 |
|  | **5-57** | B14 | VDEMFPEGAGPYVDLDEAGGSTGLLMDLAANEKAVHADFFNDFEDLFDDDDIQ | 0.7267 | 0.5694 | 0.8196 | 0.7377 | 0.6284 | 1.4711 | **0.8255** | 0.1094 |
|  | **4-57** | B15 | AVDEMFPEGAGPYVDLDEAGGSTGLLMDLAANEKAVHADFFNDFEDLFDDDDIQ | 0.9541 | 0.7847 | 1.2530 | 0.6698 | 0.9021 | 1.7326 | **1.0494** | 0.1294 |
|  | **3-57** | B16 | PAVDEMFPEGAGPYVDLDEAGGSTGLLMDLAANEKAVHADFFNDFEDLFDDDDIQ | 0.9326 | 0.5685 | 1.1238 | 0.6693 | 0.8859 | 1.9068 | **1.0145** | 0.1598 |
|  | **2-57** | B17 | KPAVDEMFPEGAGPYVDLDEAGGSTGLLMDLAANEKAVHADFFNDFEDLFDDDDIQ | 1.0251 | 0.7899 | 1.2655 | 0.6911 | 0.9125 | 1.8572 | **1.0902** | 0.1418 |
|  | **1-57** | B18 | MKPAVDEMFPEGAGPYVDLDEAGGSTGLLMDLAANEKAVHADFFNDFEDLFDDDDIQ | 0.8825 | 0.5364 | 1.1209 | 0.6103 | 0.7784 | 1.7824 | **0.9518** | 0.1522 |

|  |  |  |  | **intensities as fold of C-CSNAP (A1)** | | | | | |  |  | |
| --- | --- | --- | --- | --- | --- | --- | --- | --- | --- | --- | --- | --- |
|  |  |  |  | **Experiment #1** | | **Experiment #2** | | **Experiment #3** | |  |  | |
|  | **Residues** | **Position** | **sequence** | **left** | **right** | **left** | **right** | **left** | **right** | **Average fold change** | **standard error** |  |
| **Ala substitution** | **D42A** | B20 | AFFNDFEDLFDDDDIQ | 0.7153 | 0.3968 | 0.8273 | 0.5519 | 0.4002 | 0.6491 | **0.5901** | 0.0578 |  |
|  | **F43A** | B21 | DAFNDFEDLFDDDDIQ |  | 0.6852 | 1.1799 | 0.6324 | 0.6435 | 1.1136 | **0.8509** | 0.0906 |  |
|  | **F44A** | *B22* | *DFANDFEDLFDDDDIQ* |  |  |  |  |  |  |  |  |  |
|  | **N45A** | B23 | DFFADFEDLFDDDDIQ | 1.1287 | 1.1187 | 1.3110 | 0.7175 | 0.8759 | 2.5246 | **1.2794** | 0.2149 |  |
|  | **D46A** | B24 | DFFNAFEDLFDDDDIQ | 0.4805 | 0.3335 | 0.6706 | 0.5720 | 0.3656 | 0.9148 | **0.5562** | 0.0720 |  |
|  | **F47A** | C 1 | DFFNDAEDLFDDDDIQ | 0.5938 | 0.4366 | 0.3052 | 0.4132 | 0.2211 | 0.3302 | **0.3834** | 0.0430 |  |
|  | **E48A** | C 2 | DFFNDFADLFDDDDIQ | 1.0396 | 1.0010 | 1.1375 | 1.2264 | 0.9509 | 1.5992 | **1.1591** | 0.0791 |  |
|  | **D49A** | C 3 | DFFNDFEALFDDDDIQ | 0.8970 | 0.9888 | 1.0558 | 1.1267 | 0.8157 | 1.4853 | **1.0616** | 0.0784 |  |
|  | **L50A** | C 4 | DFFNDFEDAFDDDDIQ | 0.6588 | 0.5638 | 0.4498 | 0.5798 | 0.4180 | 0.5309 | **0.5335** | 0.0295 |  |
|  | **F51A** | C 5 | DFFNDFEDLADDDDIQ | 0.5270 | 0.4789 | 0.3212 | 0.4373 | 0.3597 | 0.2263 | **0.3917** | 0.0369 |  |
|  | **D52A** | C 6 | DFFNDFEDLFADDDIQ | 0.8300 | 0.9865 | 0.9133 | 0.9970 | 0.9065 | 1.3812 | **1.0024** | 0.0651 |  |
|  | **D53A** | C 7 | DFFNDFEDLFDADDIQ | 0.8197 | 0.9576 | 0.8910 | 0.8543 | 0.6941 | 1.2061 | **0.9038** | 0.0573 |  |
|  | **D54A** | C 8 | DFFNDFEDLFDDADIQ | 0.8237 | 0.8933 | 0.9646 | 0.8821 | 0.8420 | 1.3786 | **0.9640** | 0.0696 |  |
|  | **D55A** | C 9 | DFFNDFEDLFDDDAIQ | 0.8583 | 0.9334 | 1.0873 | 0.8962 | 0.8173 | 1.4976 | **1.0150** | 0.0847 |  |
|  | **I56A** | C10 | DFFNDFEDLFDDDDAQ | 1.0099 | 0.9509 | 1.2619 | 0.9797 | 1.0598 | 1.5728 | **1.1392** | 0.0799 |  |
|  | **Q57A** | C11 | DFFNDFEDLFDDDDIA | 1.0146 | 1.0043 | 1.1526 | 0.8897 | 1.0460 | 1.4871 | 1.0990 | 0.0693 |  |

|  |  |  |  | **intensities as fold of C-CSNAP (A1)** | | | | | |  |  |
| --- | --- | --- | --- | --- | --- | --- | --- | --- | --- | --- | --- |
|  |  |  |  | **Experiment #1** | | **Experiment #2** | | **Experiment #3** | |  |  |
|  | **Residues** | **Position** | **sequence** | **left** | **right** | **left** | **right** | **left** | **right** | **Average fold change** | **standard error** |
| **Phe to Trp substitution** | **F43W** | C12 | DWFNDFEDLFDDDDIQ | 0.7480 | 0.6322 | 0.7712 | 0.6646 | 0.5414 | 1.2960 | **0.7756** | 0.0894 |
|  | **F44W** | C13 | DFWNDFEDLFDDDDIQ | 0.7305 | 0.7333 | 0.9344 | 0.6335 | 0.6264 | 1.2456 | **0.8173** | 0.0792 |
|  | **F47W** | C14 | DFFNDWEDLFDDDDIQ | 0.7318 | 0.8003 | 1.1058 | 0.7119 | 0.6784 | 1.6358 | **0.9440** | 0.1243 |
|  | **F51W** | C15 | DFFNDFEDLWDDDDIQ | 0.7090 | 0.7159 | 0.9025 | 0.6394 | 0.5729 | 1.3556 | **0.8159** | 0.0955 |
|  | **F43,44W** | C16 | DWWNDFEDLFDDDDIQ | 0.6710 | 0.6146 | 0.7415 | 0.5135 | 0.5944 | 1.3209 | **0.7427** | 0.0978 |
|  | **F43,47W** | C17 | DWFNDWEDLFDDDDIQ | 0.7267 | 0.6190 | 0.9251 | 0.5630 | 0.6452 | 1.2965 | **0.7959** | 0.0919 |
|  | **F43,51W** | C18 | DWFNDFEDLWDDDDIQ | 0.5251 | 0.4325 | 0.6513 | 0.4224 | 0.4396 | 0.8495 | **0.5534** | 0.0563 |
|  |  | C19 | DFWNDWEDLTDDDDIQ | 0.4121 | 0.3096 | 0.4222 | 0.2807 | 0.2950 | 0.5655 |  | 0.0363 |
|  |  | C20 | DFWNDTEDLWDDDDIQ | 0.4355 | 0.3463 | 0.4061 | 0.2526 | 0.2267 | 0.5701 |  | 0.0423 |
|  |  | C21 | DFTNDWEDLWDDDDIQ | 0.5014 | 0.4443 | 0.5007 | 0.3448 | 0.2443 | 0.7433 |  | 0.0565 |
|  | **F44,47,51W** | C22 | DFWNDWEDLWDDDDIQ | 0.6025 | 0.5312 | 0.8097 | 0.4726 | 0.3206 | 0.9307 | **0.6112** | 0.0748 |
|  | **F43,47,51W** | C23 | DWFNDWEDLWDDDDIQ | 0.4545 | 0.4111 | 0.5715 | 0.3429 | 0.2895 | 0.8949 | **0.4941** | 0.0730 |
|  |  | C24 | DWWNDWEDLTDDDDIQ | 0.3414 | 0.2419 | 0.3475 | 0.2058 | 0.1376 | 0.3791 |  | 0.0317 |
|  |  | D 1 | DWWNDTEDLWDDDDIQ | 0.4334 | 0.4366 | 0.4188 | 0.4571 | 0.2579 | 0.4898 |  | 0.0270 |
|  | **F43,44,47,51W** | D 2 | DWWNDWEDLWDDDDIQ | 0.6079 | 1.0010 | 0.6768 | 0.6820 | 0.3822 | 0.7303 | **0.6800** | 0.0666 |

|  |  |  |  | **intensities as fold of C-CSNAP (A1)** | | | | | |  |  |
| --- | --- | --- | --- | --- | --- | --- | --- | --- | --- | --- | --- |
|  |  |  |  | **Experiment #1** | | **Experiment #2** | | **Experiment #3** | |  |  |
|  | **Residues** | **Position** | **sequence** | **left** | **right** | **left** | **right** | **left** | **right** | **Average fold change** | **standard error** |
| **Asp to Glu substitution** | **D42E** | D 3 | EFFNDFEDLFDDDDIQ | 1.0357 | 0.9888 | 1.1725 | 1.1598 | 1.0056 | 1.6854 | **1.1746** | 0.0874 |
|  | **D46E** | D 4 | DFFNEFEDLFDDDDIQ | 0.8536 | 0.5638 | 1.0644 | 1.1167 | 0.8775 | 1.6772 | **1.0255** | 0.1246 |
|  | **D49E** | D 5 | DFFNDFEELFDDDDIQ | 0.7959 | 0.4789 | 0.9178 | 0.8968 | 0.7478 | 1.0143 | **0.8086** | 0.0623 |
|  | **D52E** | D 6 | DFFNDFEDLFEDDDIQ | 0.8285 | 0.9865 | 1.0516 | 1.0485 | 0.7573 | 1.2132 | **0.9809** | 0.0551 |
|  | **D53E** | D 7 | DFFNDFEDLFDEDDIQ | 0.7780 | 0.9576 | 0.8556 | 0.8887 | 0.7330 | 1.1921 | **0.9008** | 0.0545 |
|  | **D54E** | D 8 | DFFNDFEDLFDDEDIQ | 0.7876 | 0.8933 | 1.0473 | 0.9273 | 0.8352 | 1.4108 | **0.9836** | 0.0758 |
|  | **D55E** | D 9 | DFFNDFEDLFDDDEIQ | 0.8464 | 0.9334 | 1.0713 | 0.8911 | 0.9076 | 1.5092 | **1.0265** | 0.0828 |
|  | **D42,46E** | D10 | EFFNEFEDLFDDDDIQ | 0.6881 | 0.9509 | 0.9437 | 0.7949 | 0.7705 | 1.1692 | **0.8862** | 0.0575 |
|  | **D42,49E** | D11 | EFFNDFEELFDDDDIQ | 0.6997 | 1.0043 | 0.7948 | 0.6865 | 0.7793 | 1.1233 | **0.8480** | 0.0588 |
|  | **D42,52E** | D12 | EFFNEFEDLFEDDDIQ | 0.6245 | 0.6322 | 0.8229 | 0.6602 | 0.6328 | 0.8425 | **0.7025** | 0.0339 |
|  | **D42,53E** | D13 | EFFNDFEDLFDEDDIQ | 0.6863 | 0.7333 | 0.7360 | 0.5930 | 0.5924 | 1.1948 | **0.7560** | 0.0748 |
|  | **D42,54E** | D14 | EFFNDFEDLFDDEDIQ | 0.6429 | 0.8003 | 0.6260 | 0.5132 | 0.5703 | 1.0834 | **0.7060** | 0.0695 |
|  | **D42,55E** | D15 | EFFNDFEDLFDDDEIQ | 0.7159 | 0.7159 | 0.7526 | 0.5223 | 0.6365 | 1.3232 | **0.7777** | 0.0932 |
|  | **D46,49E** | D16 | DFFNEFEELFDDDDIQ | 0.7165 | 0.6146 | 0.6827 | 0.4847 | 0.6545 | 1.1967 | **0.7250** | 0.0816 |
|  | **D46,52E** | D17 | DFFNEFEDLFEDDDIQ | 0.8064 | 0.6190 | 0.7123 | 0.5455 | 0.6836 | 1.2263 | **0.7655** | 0.0808 |
|  | **D46,53E** | D18 | DFFNEFEDLFDEDDIQ | 0.5950 | 0.4325 | 0.5351 | 0.3850 | 0.5637 | 0.8238 | **0.5559** | 0.0513 |
|  | **D46,54E** | D19 | DFFNEFEDLFDDEDIQ | 0.7484 | 0.3096 | 0.5872 | 0.4063 | 0.6100 | 1.0371 | **0.6165** | 0.0861 |
|  | **D46,55E** | D20 | DFFNEFEDLFDDDEIQ | 0.8459 | 0.3463 | 0.8117 | 0.4948 | 0.7747 | 1.4059 | **0.7799** | 0.1217 |
|  | **D49,52E** | D21 | DFFNDFEELFEDDDIQ | 0.7022 | 0.4443 | 0.6550 | 0.3782 | 0.5280 | 1.0931 | **0.6335** | 0.0854 |
|  | **D49,53E** | D22 | DFFNDFEELFDEDDIQ | 0.7823 | 0.5312 | 0.7453 | 0.3615 | 0.5394 | 1.0255 | **0.6642** | 0.0783 |
|  | **D49,54E** | D23 | DFFNDFEELFDDEDIQ | 0.6117 | 0.4111 | 0.6919 | 0.3381 | 0.4764 | 0.9656 | **0.5825** | 0.0760 |
|  | **D49,55E** | D24 | DFFNDFEELFDDDEIQ | 0.6003 | 0.2419 | 0.6850 | 0.2796 | 0.5960 | 0.8055 | **0.5347** | 0.0752 |
|  | **D52,53E** | E 1 | DFFNDFEDLFEEDDIQ | 0.7235 | 0.6124 | 0.7260 | 0.6301 | 0.6108 | 0.8456 | **0.6914** | 0.0307 |
|  | **D52,54E** | E 2 | DFFNDFEDLFEDEDIQ | 0.8740 | 0.8747 | 0.8553 | 0.8033 | 0.7322 | 1.0770 | **0.8694** | 0.0385 |
|  | **D52,55E** | E 3 | DFFNDFEDLFEDDEIQ | 0.8908 | 0.8227 | 0.9347 | 0.8530 | 0.7138 | 1.1122 | **0.8879** | 0.0443 |
|  | **D42,46,49E** | E 4 | EFFNEFEELFDDDDIQ | 0.8107 | 0.7670 | 0.9323 | 0.7647 | 0.7180 | 1.0317 | **0.8374** | 0.0400 |
|  | **D42,46,52E** | E 5 | EFFNEFEDLFEDDDIQ | 0.7945 | 0.7698 | 0.9330 | 0.8675 | 0.7198 | 1.1918 | **0.8794** | 0.0568 |
|  | **D42,46,53E** | E 6 | EFFNEFEDLFDEDDIQ | 0.6668 | 0.6126 | 0.6073 | 0.7637 | 0.6087 | 0.9415 | **0.7001** | 0.0442 |
|  | **D42,46,54E** | E 7 | EFFNEFEDLFDDEDIQ | 0.7018 | 0.6534 | 0.6287 | 0.7652 | 0.5225 | 0.7117 | **0.6639** | 0.0280 |
|  | **D42,46,55E** | E 8 | EFFNEFEDLFDDDEIQ | 0.6904 | 0.7236 | 0.6842 | 0.7651 | 0.6028 | 1.1033 | **0.7616** | 0.0586 |
|  | **D42,49,52E** | E 9 | EFFNDFEELFEDDDIQ | 0.6181 | 0.6382 | 0.6702 | 0.6566 | 0.5823 | 0.9039 | **0.6782** | 0.0383 |
|  | **D42,49,53E** | E10 | EFFNDFEELFDEDDIQ | 0.6598 | 0.6438 | 0.6418 | 0.6909 | 0.5870 | 0.9437 | **0.6945** | 0.0422 |
|  | **D42,49,54E** | E11 | EFFNDFEELFDDEDIQ | 0.6321 | 0.6453 | 0.6032 | 0.6505 | 0.5667 | 1.0122 | **0.6850** | 0.0544 |
|  | **D42,49,55E** | E12 | EFFNDFEELFDDDEIQ | 0.5595 | 0.6791 | 0.5933 | 0.6229 | 0.5572 | 1.0045 | **0.6694** | 0.0568 |
|  | **D46,49,52E** | E13 | DFFNEFEELFEDDDIQ | 0.5857 | 0.6912 | 0.5198 | 0.5904 | 0.5662 | 1.0020 | **0.6592** | 0.0590 |
|  | **D46,49,53E** | E14 | DFFNEFEELFDEDDIQ | 0.6274 | 0.6237 | 0.5086 | 0.5062 | 0.4905 | 0.9614 | **0.6197** | 0.0594 |
|  | **D46,49,54E** | E15 | DFFNEFEELFDDEDIQ | 0.5626 | 0.5970 | 0.4144 | 0.4595 | 0.4223 | 0.9523 | **0.5680** | 0.0675 |
|  | **D46,49,55E** | E16 | DFFNEFEELFDDDEIQ | 0.6803 | 0.6840 | 0.5085 | 0.5106 | 0.5731 | 1.2330 | **0.6983** | 0.0911 |
|  | **D49,52,53E** | E17 | DFFNDFEELFEEDDIQ | 0.6672 | 0.6416 | 0.4739 | 0.4322 | 0.4613 | 1.0471 | **0.6206** | 0.0770 |
|  | **D49,52,54E** | E18 | DFFNDFEELFEDEDIQ | 0.6348 | 0.6603 | 0.5139 | 0.4145 | 0.5040 | 1.0584 | **0.6310** | 0.0760 |
|  | **D49,52,55E** | E19 | DFFNDFEELFEDDEIQ | 0.6266 | 0.6743 | 0.5157 | 0.3958 | 0.5023 | 1.1542 | **0.6448** | 0.0894 |

|  |  |  | **intensities as fold of C-CSNAP (A1)** | | | | | |  |  |
| --- | --- | --- | --- | --- | --- | --- | --- | --- | --- | --- |
|  |  |  | **Experiment #1** | | **Experiment #2** | | **Experiment #3** | |  |  |
| **Residues** | **Position** | **sequence** | **left** | **right** | **left** | **right** | **left** | **right** | **Average fold change** | **standard error** |
| **D52,53,55E** | E21 | DFFNDFEDLFEEDEIQ | 0.7298 | 0.7953 | 0.6472 | 0.4018 | 0.4974 | 1.0785 | **0.6917** | 0.0797 |
| **D52,53,56E** | E22 | DFFNDFEDLFEDEEIQ | 0.6772 | 0.6462 | 0.5263 | 0.3909 | 0.3890 | 0.9867 | **0.6027** | 0.0747 |
| **D42,46,49,52E** | E23 | EFFNEFEELFEDDDIQ | 0.5632 | 0.5559 | 0.5443 | 0.2699 | 0.4038 | 0.7635 | **0.5168** | 0.0556 |
| **D42,46,49,53E** | E24 | EFFNEFEELFDEDDIQ | 0.4571 | 0.4501 | 0.4095 | 0.1934 | 0.2796 | 0.4947 | **0.3807** | 0.0394 |
| **D42,46,49,54E** | F 1 | EFFNEFEELFDDEDIQ | 0.6032 | 0.4702 | 0.6585 | 0.4572 | 0.3892 | 0.6773 | **0.5426** | 0.0398 |
| **D42,46,49,55E** | F 2 | EFFNEFEELFDDDEIQ | 0.5842 | 0.4497 | 0.7078 | 0.5485 | 0.4676 | 0.8046 | **0.5938** | 0.0463 |
| **D42,49,52,53E** | F 3 | EFFNDFEELFEEDDIQ | 0.7400 | 0.6877 | 0.7165 | 0.6684 | 0.6404 | 0.9014 | **0.7257** | 0.0310 |
| **D42,49,52,54E** | F 4 | EFFNDFEELFEDEDIQ | 0.7293 | 0.5995 | 0.6777 | 0.7412 | 0.7132 | 0.7992 | **0.7100** | 0.0224 |
| **D42,49,52,55E** | F 5 | EFFNDFEELFEDDEIQ | 0.7291 | 0.7184 | 0.6348 | 0.8121 | 0.6667 | 0.9564 | **0.7529** | 0.0389 |
| **D42,52,53,54E** | F 6 | EFFNDFEDLFEEEDIQ | 0.6977 | 0.6693 | 0.6617 | 0.8612 | 0.6170 | 0.9522 | **0.7432** | 0.0442 |
| **D42,52,53,55E** | F 7 | EFFNDFEDLFEEDEIQ | 0.6483 | 0.6541 | 0.5699 | 0.8034 | 0.4862 | 0.7392 | **0.6502** | 0.0379 |
| **D46,49,52,53E** | F 8 | DFFNEFEELFEEDDIQ | 0.5470 | 0.5427 | 0.5425 | 0.7389 | 0.4465 | 0.7133 | **0.5885** | 0.0378 |
| **D46,49,52,54E** | F 9 | DFFNEFEELFEDEDIQ | 0.5521 | 0.6093 | 0.4882 | 0.6717 | 0.4407 | 0.7987 | **0.5934** | 0.0434 |
| **D46,49,52,55E** | F10 | DFFNEFEELFEDDEIQ | 0.5947 | 0.5446 | 0.5050 | 0.6442 | 0.5120 | 0.9513 | **0.6253** | 0.0560 |
| **D49,52,53,54E** | F11 | DFFNDFEELFEEEDIQ | 0.4979 | 0.5786 | 0.4861 | 0.5953 | 0.4665 | 1.1098 | **0.6224** | 0.0815 |
| **D49,52,53,55E** | F12 | DFFNDFEELFEEDEIQ | 0.6235 | 0.6239 | 0.6244 | 0.6510 | 0.5718 | 1.1027 | **0.6996** | 0.0664 |
| **D52,53,54,55E** | F13 | DFFNDFEDLFEEEEIQ | 0.6652 | 0.7735 | 0.6203 | 0.6801 | 0.4511 | 1.1628 | **0.7255** | 0.0797 |
| **D42,46,49,52,53E** | F14 | EFFNEFEELFEEDDIQ | 0.5824 | 0.6448 | 0.5356 | 0.6762 | 0.4988 | 1.2346 | **0.6954** | 0.0907 |
| **D42,46,49,52,54E** | F15 | EFFNEFEELFEDEDIQ | 0.5603 | 0.6417 | 0.3839 | 0.5295 | 0.3747 | 1.0901 | **0.5967** | 0.0877 |
| **D42,46,49,52,55E** | F16 | EFFNEFEELFEDDEIQ | 0.5360 | 0.5076 | 0.3560 | 0.4561 | 0.3423 | 0.9278 | **0.5210** | 0.0714 |
| **D46,49,52,53,54E** | F17 | DFFNEFEELFEEEDIQ | 0.6274 | 0.6443 | 0.3822 | 0.4943 | 0.3731 | 0.9133 | **0.5724** | 0.0677 |
| **D46,49,52,54,55E** | F18 | DFFNEFEELFEDEEIQ | 0.5918 | 0.6919 | 0.4223 | 0.4731 | 0.4042 | 0.9977 | **0.5968** | 0.0749 |
| **D46,49,52,53,55E** | F19 | DFFNEFEELFEEDEIQ | 0.5735 | 0.6012 | 0.3686 | 0.3967 | 0.3867 | 1.0796 | **0.5677** | 0.0900 |
| **D46,49,53,54,55E** | F20 | DFFNEFEELFDEEEIQ | 0.5770 | 0.6620 | 0.4975 | 0.3789 | 0.4436 | 1.1622 | **0.6202** | 0.0945 |
| **D49,52,53,54,55E** | F21 | DFFNDFEELFEEEEIQ | 0.6322 | 0.7458 | 0.6012 | 0.4106 | 0.4591 | 1.1458 | **0.6658** | 0.0882 |
| **D42,46,49,52,53,55E** | F22 | EFFNEFEELFEEEDIQ | 0.3674 | 0.4485 | 0.3610 | 0.2643 | 0.2678 | 0.7359 | **0.4075** | 0.0584 |
| **D42,46,49,53,54,55E** | F23 | EFFNEFEELFDEEEIQ | 0.3404 | 0.4285 | 0.3045 | 0.2166 | 0.2677 | 0.6149 | **0.3621** | 0.0477 |
| **D42,46,49,52,54,55E** | F24 | EFFNEFEELFEDEEIQ | 0.2454 | 0.2454 | 0.2813 | 0.1450 | 0.2728 | 0.3732 | **0.2605** | 0.0245 |
| **D42,46,49,52,53, 54E** | G 1 | EFFNEFEELFEEEDIQ | 0.5365 | 0.4739 | 0.7346 | 0.4854 | 0.3913 | 0.7931 | **0.5691** | 0.0530 |
| **D46,49,52,53,54,55E** | G 2 | DFFNEFEELFEEEEIQ | 0.6040 | 0.5232 | 0.6112 | 0.5498 | 0.3361 | 0.6577 | **0.5470** | 0.0379 |
| **D42,49,52,53,54,55E** | G 3 | EFFNDFEELFEEEEIQ | 0.6514 | 0.5201 | 0.5791 | 0.6012 | 0.5352 | 0.7925 | **0.6132** | 0.0332 |
| **D42,46,49,52,53,54,56E** | G 4 | EFFNEFEELFEEEEIQ | 0.6680 | 0.5203 | 0.5217 | 0.5922 | 0.5948 | 0.6569 | **0.5923** | 0.0211 |

|  |  |  |  | **intensities as fold of C-CSNAP (A1)** | | | | | |  |  |
| --- | --- | --- | --- | --- | --- | --- | --- | --- | --- | --- | --- |
|  |  |  |  | **Experiment #1** | | **Experiment #2** | | **Experiment #3** | |  |  |
|  | **Residues** | **Position** | **sequence** | **left** | **right** | **left** | **right** | **left** | **right** | **Average fold change** | **standard error** |
| **d-AA substitution** | **D42d** | G 5 | dFFNDFEDLFDDDDIQ | 1.0186 | 1.0304 | 1.0572 | 1.1230 | 1.0765 | 1.6368 | **1.1571** | 0.0793 |
|  | **F43f** | G 6 | DfFNDFEDLFDDDDIQ | 0.9777 | 0.9563 | 1.0095 | 1.2250 | 1.0495 | 1.6370 | **1.1425** | 0.0869 |
|  | **F44f** | G 7 | DFfNDFEDLFDDDDIQ | 0.9173 | 0.8726 | 1.0694 | 1.1973 | 0.7850 | 1.6330 | **1.0791** | 0.1029 |
|  | **N45n** | G 8 | DFFnDFEDLFDDDDIQ | 0.8510 | 0.9475 | 0.9340 | 1.0358 | 0.6499 | 1.4924 | **0.9851** | 0.0936 |
|  | **D4d6** | G 9 | DFFNdFEDLFDDDDIQ | 0.7545 | 0.8114 | 0.8371 | 1.0682 | 0.6478 | 1.6942 | **0.9689** | 0.1271 |
|  | **F47f** | G10 | DFFNDfEDLFDDDDIQ | 0.3825 | 0.3651 | 0.2798 | 0.5784 | 0.3292 | 0.8571 | **0.4653** | 0.0724 |
|  | **E48e** | G11 | DFFNDeDLFDDDDIQ | 0.2751 | 0.1932 | 0.1428 | 0.3200 | 0.2138 | 0.3580 | **0.2505** | 0.0272 |
|  | **D49d** | G12 | DFFNDFEdLFDDDDIQ | 0.8322 | 1.0953 | 1.0440 | 1.0098 | 0.9934 | 2.4591 | **1.2390** | 0.2014 |
|  | **L50l** | G13 | DFFNDFEDlFDDDDIQ | 0.8436 | 1.2749 | 1.3309 | 1.3026 | 1.2027 | 3.0686 | **1.5039** | 0.2624 |
|  | **F51f** | G14 | DFFNDFEDLfDDDDIQ | 1.0433 | 1.2008 | 1.1324 | 1.2275 | 1.1933 | 2.7968 | **1.4324** | 0.2239 |
|  | **D52d** | G15 | DFFNDFEDLFdDDDIQ | 0.8389 | 1.1288 | 1.0146 | 0.9338 | 0.8657 | 2.5198 | **1.2169** | 0.2156 |
|  | **D53d** | G16 | DFFNDFEDLFDdDDIQ | 0.8478 | 1.0496 | 1.0697 | 0.9110 | 0.9009 | 2.6173 | **1.2327** | 0.2280 |
|  | **D54d** | G17 | DFFNDFEDLFDDdDIQ | 1.0370 | 1.1329 | 1.2073 | 1.0271 | 1.2671 | 2.6062 | **1.3796** | 0.2027 |
|  | **D55d** | G18 | DFFNDFEDLFDDDdIQ | 0.9093 | 1.1427 | 1.0152 | 0.8865 | 1.1519 | 2.5972 | **1.2838** | 0.2177 |
|  | **I56i** | G19 | DFFNDFEDLFDDDDiQ | 0.8875 | 0.8791 | 0.9892 | 0.7619 | 0.9690 | 1.7766 | **1.0439** | 0.1226 |
|  | **Q57q** | G20 | DFFNDFEDLFDDDDIq | 0.7774 | 0.8333 | 0.9631 | 0.6765 | 1.0552 | 1.9690 | **1.0457** | 0.1573 |

|  |  |  |  | **intensities as fold of C-CSNAP (A1)** | | | | | | |  |  |
| --- | --- | --- | --- | --- | --- | --- | --- | --- | --- | --- | --- | --- |
|  |  |  |  | **Experiment #1** | | | **Experiment #2** | | **Experiment #3** | |  |  |
|  | **Residues** | **Position** | **sequence** | | **left** | **right** | **left** | **right** | **left** | **right** | **Average fold change** | **standard error** |
| **Non-proteogenic AA substitution** | **N-Met-D** | H13 | N-Me-Asp-FFNDFEDLFDDDDIQ | | 0.5765 | 0.5803 | 0.8311 | 0.8958 | 0.5199 | 2.3597 | **0.9606** | 0.2340 |
|  | **3x(LL-Aib)** | H14 | LL-Aib-LL-Aib-LL-Aib-GGG-DFFNDFEDLFDDDDIQ | | 0.7859 | 0.6813 | 1.0090 | 1.0967 | 0.8398 | 2.8362 | **1.2081** | 0.2706 |
|  | **+Aib(46,52,58)** | H15 | DFFN-Aib-FEDLF-Aib-DDDIQ-Aib | | 0.3458 | 0.3290 | 0.2291 | 0.4652 | 0.2799 | 1.0762 | **0.4542** | 0.1049 |
|  | **+Aib(45,50,56)** | H16 | DFF-Aib-DFED-Aib-FDDDI-Aib-Q | | 0.3868 | 0.3074 | 0.3253 | 0.4113 | 0.4290 | 1.0982 | **0.4930** | 0.1001 |
|  | **+Aib(46,52,57)** | H17 | DFFN-Aib-FEDLF-Aib-DDDI-Aib | | 0.3158 | 0.2689 | 0.1472 | 0.2740 | 0.2292 | 0.8343 | **0.3449** | 0.0821 |
|  | **+Aib(54,58)** | H18 | DFFNDFEDLFDD-Aib-DIQ-Aib | | 0.3071 | 0.2334 | 0.1973 | 0.2444 | 0.2003 | 0.6096 | **0.2987** | 0.0525 |
|  | **+Aib(42,48,54)** | H19 | Aib-FFNDF-Aib-DLFDD-Aib-DIQ | | 0.3903 | 0.3767 | 0.4743 | 0.3471 | 0.4598 | 0.9425 | **0.4984** | 0.0743 |
