# Supplementary material for: The C-terminal tail of CSNAP attenuates the CSN complex
Source: Life Sci Alliance. 2023 Jul 17;6(10):e202201634. doi: 10.26508/lsa.202201634 (PMC10355216; doi:10.26508/lsa.202201634)
Supplement: Supplementary file 2 [file LSA-2022-01634_TableS2.docx]

**Table 2 - Peptide array data for rCSN^ΔCSNAP^ used in Fig.S3.**

|  |  |  |  | **intensities as fold of C-CSNAP (A1)** | | | | | | | | | |  |  |
| --- | --- | --- | --- | --- | --- | --- | --- | --- | --- | --- | --- | --- | --- | --- | --- |
|  |  |  |  | Experiment #1 | | Experiment #2 | | Experiment #3 | | Experiment #4 | | Experiment #5 | |  |  |
|  | **Residues** | **Position** | **sequence** | **left** | **right** | **left** | **right** | **left** | **right** | **left** | **right** | **left** | **right** | **Average fold change** | **standard error** |
| **Elongation** | **42-57** | A 1 | DFFNDFEDLFDDDDIQ | 1.0000 | 1.0000 | 1.0000 | 1.0000 | 1.0000 | 1.0000 | 1.0000 | 1.0000 | 1.0000 | 1.0000 | **1.0000** | 0.0000 |
|  | **41-57** | A 2 | ADFFNDFEDLFDDDDIQ | 0.9340 | 0.8382 | 0.8006 | 0.8686 | 0.9346 | 0.9679 | 0.9919 | 0.8037 | 0.9158 | 1.0026 | **0.9058** | 0.0248 |
|  | **40-57** | A 3 | HADFFNDFEDLFDDDDIQ | 0.8915 | 0.7222 | 0.6304 | 0.7754 | 0.8953 | 0.8705 | 0.8776 | 0.7546 | 0.8203 | 0.9772 | **0.8215** | 0.0339 |
|  | **39-57** | A 4 | VHADFFNDFEDLFDDDDIQ | 0.9545 | 0.9570 | 0.7290 | 0.9039 | 1.0423 | 0.9394 | 1.0909 | 0.8197 | 0.9237 | 1.1911 | **0.9552** | 0.0438 |
|  | **38-57** | A 5 | AVHADFFNDFEDLFDDDDIQ | 1.0704 | 1.0324 | 0.8260 | 0.9847 | 1.1492 | 0.9710 | 1.3757 | 0.9266 | 0.9868 | 1.2931 | **1.0616** | 0.0560 |
|  | **37-57** | A 6 | KAVHADFFNDFEDLFDDDDIQ | 1.0180 | 0.8222 | 0.7195 | 0.8361 | 1.0035 | 0.8939 | 1.1446 | 0.7915 | 0.8626 | 1.0869 | **0.9179** | 0.0461 |
|  | **36-57** | A 7 | EKAVHADFFNDFEDLFDDDDIQ | 1.2324 | 1.0880 | 0.9641 | 1.1704 | 1.1393 | 1.0820 | 1.4517 | 1.0149 | 0.9408 | 1.2278 | **1.1311** | 0.0504 |
|  | **35-57** | A 8 | NEKAVHADFFNDFEDLFDDDDIQ | 1.1151 | 0.8856 | 0.7737 | 0.8488 | 0.9170 | 0.9160 | 1.2162 | 0.9038 | 0.8786 | 1.1254 | **0.9580** | 0.0476 |
|  | **34-57** | A 9 | ANEKAVHADFFNDFEDLFDDDDIQ | 1.3307 | 1.2174 | 0.9291 | 0.9777 | 1.0348 | 1.0707 | 1.4571 | 1.1675 | 0.9405 | 1.4020 | **1.1527** | 0.0645 |
|  | **33-57** | A10 | AANEKAVHADFFNDFEDLFDDDDIQ | 1.2867 | 0.9129 | 0.8503 | 0.8335 | 0.9117 | 0.9534 | 1.1456 | 0.8602 | 0.8540 | 1.2626 | **0.9871** | 0.0587 |
|  | **32-57** | A11 | LAANEKAVHADFFNDFEDLFDDDDIQ | 1.5955 | 1.1454 | 1.0946 | 1.0720 | 1.0599 | 1.0910 | 1.5013 | 0.9972 | 0.8862 | 1.3474 | **1.1791** | 0.0759 |
|  | **31-57** | A12 | DLAANEKAVHADFFNDFEDLFDDDDIQ | 1.3892 | 0.9987 | 0.8647 | 0.8997 | 0.9675 | 1.0358 | 1.3170 | 0.8747 | 0.7425 | 1.3154 | **1.0405** | 0.0743 |
|  | **30-57** | A13 | MDLAANEKAVHADFFNDFEDLFDDDDIQ | 1.5219 | 1.1652 | 0.8490 | 1.0223 | 1.0021 | 1.1023 | 1.5483 | 1.0247 | 0.8770 | 1.3639 | **1.1476** | 0.0835 |
|  | **29-57** | A14 | LMDLAANEKAVHADFFNDFEDLFDDDDIQ | 1.2397 | 0.9718 | 0.9356 | 0.8443 | 0.9380 | 1.0036 | 1.6229 | 0.9885 | 0.7703 | 1.4114 | **1.0726** | 0.0894 |
|  | **28-57** | A15 | LLMDLAANEKAVHADFFNDFEDLFDDDDIQ | 1.4128 | 1.2089 | 1.1335 | 0.9772 | 1.0430 | 1.1236 | 1.8069 | 1.0743 | 0.8510 | 1.4025 | **1.2034** | 0.0914 |
|  | **27-57** | A16 | GLLMDLAANEKAVHADFFNDFEDLFDDDDIQ | 1.5392 | 1.1038 | 1.2475 | 0.9001 | 0.9759 | 1.0563 | 2.0187 | 1.0386 | 0.8588 | 1.4287 | **1.2168** | 0.1191 |
|  | **26-57** | A17 | TGLLMDLAANEKAVHADFFNDFEDLFDDDDIQ | 1.6681 | 1.3188 | 1.3133 | 1.0845 | 1.0518 | 1.1920 | 2.4550 | 1.3210 | 0.9187 | 1.4705 | **1.3794** | 0.1449 |
|  | **25-57** | A18 | STGLLMDLAANEKAVHADFFNDFEDLFDDDDIQ | 1.4969 | 1.3191 | 1.3198 | 1.0336 | 1.0165 | 1.4500 | 2.3203 | 1.2913 | 0.7992 | 1.5158 | **1.3562** | 0.1370 |
|  | **24-57** | A19 | GSTGLLMDLAANEKAVHADFFNDFEDLFDDDDIQ | 1.2612 | 1.1221 | 1.0773 | 0.8276 | 0.8791 | 1.2086 | 2.0786 | 1.0119 | 0.7253 | 1.3245 | **1.1516** | 0.1263 |
|  | **23-57** | A20 | GGSTGLLMDLAANEKAVHADFFNDFEDLFDDDDIQ | 1.3117 | 1.1222 | 1.0834 | 0.8072 | 0.7975 | 1.2565 | 2.0876 | 1.0314 | 0.8088 | 1.4308 | **1.1737** | 0.1299 |
|  | **22-57** | A21 | AGGSTGLLMDLAANEKAVHADFFNDFEDLFDDDDIQ | 1.2871 | 1.3927 | 1.1925 | 0.9283 | 0.7702 | 1.3845 | 2.3242 | 1.3779 | 0.8259 | 1.4735 | **1.2957** | 0.1472 |
|  | **21-57** | A22 | EAGGSTGLLMDLAANEKAVHADFFNDFEDLFDDDDIQ | 1.1910 | 1.2992 | 1.2683 | 0.9326 | 0.8391 | 1.4541 | 2.5073 | 1.2976 | 0.8918 | 1.4824 | **1.3163** | 0.1587 |
|  | **20-57** | A23 | DEAGGSTGLLMDLAANEKAVHADFFNDFEDLFDDDDIQ | 1.2960 | 1.3523 | 1.2314 | 0.9358 | 1.0542 | 1.4427 | 2.6102 | 1.4179 | 0.9555 | 1.6059 | **1.3902** | 0.1603 |
|  | **19-57** | A24 | LDEAGGSTGLLMDLAANEKAVHADFFNDFEDLFDDDDIQ | 1.0560 | 1.3430 | 1.0122 | 0.7947 | 0.9196 | 1.3489 | 1.8305 | 1.1372 | 0.7873 | 1.4042 | **1.1634** | 0.1078 |
|  | **18-57** | B 1 | DLDEAGGSTGLLMDLAANEKAVHADFFNDFEDLFDDDDIQ | 1.1701 | 0.8024 | 0.7881 | 0.7204 | 0.8978 | 0.9246 | 1.0216 | 0.8288 | 1.0384 | 0.9340 | **0.9126** | 0.0453 |
|  | **17-57** | B 2 | VDLDEAGGSTGLLMDLAANEKAVHADFFNDFEDLFDDDDIQ | 0.9249 | 0.7067 | 0.6646 | 0.7027 | 0.8807 | 0.9441 | 0.8575 | 0.6609 | 0.9262 | 0.9530 | **0.8221** | 0.0410 |
|  | **16-57** | B 3 | YVDLDEAGGSTGLLMDLAANEKAVHADFFNDFEDLFDDDDIQ | 1.0639 | 0.8204 | 0.7115 | 0.7863 | 1.0175 | 1.0016 | 1.0219 | 0.8208 | 0.9814 | 0.9900 | **0.9215** | 0.0411 |
|  | **15-57** | B 4 | PYVDLDEAGGSTGLLMDLAANEKAVHADFFNDFEDLFDDDDIQ | 1.0821 | 1.0550 | 0.7990 | 1.0247 | 1.0710 | 1.1542 | 1.0674 | 0.8606 | 1.0372 | 1.0949 | **1.0246** | 0.0365 |
|  | **14-57** | B 5 | GPYVDLDEAGGSTGLLMDLAANEKAVHADFFNDFEDLFDDDDIQ | 1.0219 | 0.9097 | 0.6634 | 0.8212 | 1.0435 | 1.0286 | 1.2319 | 0.8811 | 1.0984 | 1.1164 | **0.9816** | 0.0549 |
|  | **13-57** | B 6 | AGPYVDLDEAGGSTGLLMDLAANEKAVHADFFNDFEDLFDDDDIQ | 1.2107 | 1.0671 | 0.8461 | 1.0785 | 1.2276 | 1.1926 | 1.3149 | 0.9524 | 1.0809 | 1.2166 | **1.1187** | 0.0473 |
|  | **12-57** | B 7 | GAGPYVDLDEAGGSTGLLMDLAANEKAVHADFFNDFEDLFDDDDIQ | 1.2594 | 0.9438 | 0.7342 | 1.0188 | 1.1051 | 1.0446 | 1.2958 | 1.0278 | 1.0261 | 1.2647 | **1.0720** | 0.0568 |
|  | **11-57** | B 8 | EGAGPYVDLDEAGGSTGLLMDLAANEKAVHADFFNDFEDLFDDDDIQ | 1.1696 | 0.8300 | 0.7047 | 0.9628 | 1.0440 | 1.0197 | 1.0960 | 0.8202 | 0.9222 | 1.1946 | **0.9764** | 0.0531 |
|  | **10-57** | B 9 | PEGAGPYVDLDEAGGSTGLLMDLAANEKAVHADFFNDFEDLFDDDDIQ | 1.2591 | 0.9085 | 0.7161 | 0.8561 | 0.9745 | 1.0090 | 1.2426 | 1.0033 | 0.9973 | 1.2412 | **1.0208** | 0.0599 |
|  | **9-57** | B10 | FPEGAGPYVDLDEAGGSTGLLMDLAANEKAVHADFFNDFEDLFDDDDIQ | 1.2621 | 0.9636 | 0.8157 | 0.9658 | 1.0123 | 1.1578 | 1.4011 | 1.0624 | 0.9371 | 1.2472 | **1.0825** | 0.0600 |
|  | **8-57** | B11 | MFPEGAGPYVDLDEAGGSTGLLMDLAANEKAVHADFFNDFEDLFDDDDIQ | 1.4345 | 0.9824 | 0.8352 | 0.9191 | 1.0009 | 1.0620 | 1.4864 | 1.0719 | 0.9100 | 1.2909 | **1.0993** | 0.0756 |
|  | **7-57** | B12 | EMFPEGAGPYVDLDEAGGSTGLLMDLAANEKAVHADFFNDFEDLFDDDDIQ | 1.4015 | 1.0722 | 0.7932 | 0.9375 | 0.9751 | 1.1526 | 1.6178 | 0.9713 | 0.8694 | 1.2663 | **1.1057** | 0.0858 |
|  | **6-57** | B13 | DEMFPEGAGPYVDLDEAGGSTGLLMDLAANEKAVHADFFNDFEDLFDDDDIQ | 1.3412 | 1.0096 | 0.9162 | 0.9974 | 0.9926 | 1.1430 | 1.9043 | 1.0516 | 0.9475 | 1.3135 | **1.1617** | 0.0996 |
|  | **5-57** | B14 | VDEMFPEGAGPYVDLDEAGGSTGLLMDLAANEKAVHADFFNDFEDLFDDDDIQ | 1.0933 | 0.8507 | 0.8461 | 0.8196 | 0.8413 | 1.0479 | 1.6383 | 0.8594 | 0.7704 | 1.2967 | **1.0064** | 0.0918 |
|  | **4-57** | B15 | AVDEMFPEGAGPYVDLDEAGGSTGLLMDLAANEKAVHADFFNDFEDLFDDDDIQ | 1.3416 | 1.0921 | 0.9768 | 0.8718 | 0.9677 | 1.1617 | 1.9437 | 1.1323 | 0.9064 | 1.3640 | **1.1758** | 0.1060 |
|  | **3-57** | B16 | PAVDEMFPEGAGPYVDLDEAGGSTGLLMDLAANEKAVHADFFNDFEDLFDDDDIQ | 1.5249 | 1.3445 | 1.2762 | 1.0731 | 1.0730 | 1.3519 | 2.2163 | 1.2713 | 0.9466 | 1.4625 | **1.3540** | 0.1177 |
|  | **2-57** | B17 | KPAVDEMFPEGAGPYVDLDEAGGSTGLLMDLAANEKAVHADFFNDFEDLFDDDDIQ | 1.4243 | 1.3529 | 1.2507 | 1.0902 | 1.0564 | 1.5602 | 2.3690 | 1.4229 | 0.9465 | 1.4250 | **1.3898** | 0.1319 |
|  | **1-57** | B18 | MKPAVDEMFPEGAGPYVDLDEAGGSTGLLMDLAANEKAVHADFFNDFEDLFDDDDIQ | 1.2248 | 1.3738 | 1.3632 | 1.0074 | 1.0455 | 1.4906 | 2.2490 | 1.4013 | 0.8718 | 1.4665 | **1.3494** | 0.1267 |

|  |  |  |  | **intensities as fold of C-CSNAP (A1)** | | | | | | | | | |  | |  | |
| --- | --- | --- | --- | --- | --- | --- | --- | --- | --- | --- | --- | --- | --- | --- | --- | --- | --- |
|  |  |  |  | Experiment #1 | | Experiment #2 | | Experiment #3 | | Experiment #4 | | Experiment #5 | |  |  | |  |
|  | **Residues** | **Position** | **sequence** | **left** | **right** | **left** | **right** | **left** | **right** | **left** | **right** | **left** | **right** | **Average fold change** | **standard error** | |  |
| **Ala substitution** | **D42A** | B20 | AFFNDFEDLFDDDDIQ | 1.0979 | 0.9031 | 1.0544 | 0.7538 | 0.8137 | 1.2598 | 1.6305 | 0.8757 | 0.7088 | 1.1033 | **1.0201** | 0.0922 | |  |
|  | **F43A** | B21 | DAFNDFEDLFDDDDIQ | 1.1488 | 1.1894 | 0.9976 | 0.7757 | 0.7627 | 1.2992 | 1.8825 | 1.0049 | 0.7896 | 1.1979 | **1.1048** | 0.1115 | |  |
|  | **F44A** | *B22* | *DFANDFEDLFDDDDIQ* | *1.1868* | *1.1872* | *1.1665* | *0.9547* | *0.9964* | *1.4898* | *2.1458* | *1.2185* | *0.8117* | *1.4844* | ***1.2642*** | *0.1253* | |  |
|  | **N45A** | B23 | DFFADFEDLFDDDDIQ | 1.2234 | 1.2827 | 0.9197 | 0.8305 | 0.8969 | 1.3851 | 1.7885 | 1.1195 | 0.7427 | 1.4160 | **1.1605** | 0.1078 | |  |
|  | **D46A** | B24 | DFFNAFEDLFDDDDIQ | 0.9379 | 1.3018 | 0.7890 | 0.8011 | 0.8833 | 1.5124 | 1.1229 | 1.0713 | 0.5533 | 1.2189 | **1.0192** | 0.0942 | |  |
|  | **F47A** | C 1 | DFFNDAEDLFDDDDIQ | 1.3792 | 1.1442 | 1.0519 | 0.9071 | 0.9492 | 1.1513 | 1.0760 | 1.0476 | 1.1326 | 0.9548 | **1.0794** | 0.0453 | |  |
|  | **E48A** | C 2 | DFFNDFADLFDDDDIQ | 1.4770 | 1.2045 | 0.8715 | 0.8941 | 1.0204 | 1.1668 | 0.9580 | 0.8360 | 1.1026 | 0.9913 | **1.0522** | 0.0645 | |  |
|  | **D49A** | C 3 | DFFNDFEALFDDDDIQ | 1.0928 | 0.7575 | 0.7001 | 0.7020 | 0.9514 | 0.9478 | 0.8226 | 0.6018 | 1.0908 | 1.0092 | **0.8676** | 0.0582 | |  |
|  | **L50A** | C 4 | DFFNDFEDAFDDDDIQ | 0.9723 | 0.7628 | 0.7375 | 0.8371 | 0.9458 | 1.0037 | 0.8964 | 0.6838 | 1.0373 | 1.0021 | **0.8879** | 0.0418 | |  |
|  | **F51A** | C 5 | DFFNDFEDLADDDDIQ | 1.1407 | 1.0286 | 0.8480 | 1.0434 | 1.1955 | 1.3061 | 1.3296 | 1.0149 | 1.0699 | 1.1555 | **1.1132** | 0.0481 | |  |
|  | **D52A** | C 6 | DFFNDFEDLFADDDIQ | 1.1802 | 1.0020 | 0.6624 | 0.8635 | 1.0096 | 1.0923 | 1.0762 | 0.8071 | 1.1118 | 1.0284 | **0.9833** | 0.0530 | |  |
|  | **D53A** | C 7 | DFFNDFEDLFDADDIQ | 1.4678 | 1.2573 | 0.8879 | 1.1748 | 1.1582 | 1.3056 | 1.2850 | 1.0135 | 1.1789 | 1.2251 | **1.1954** | 0.0531 | |  |
|  | **D54A** | C 8 | DFFNDFEDLFDDADIQ | 1.5364 | 1.2305 | 0.8679 | 1.1284 | 1.1434 | 1.2167 | 1.4504 | 0.9721 | 1.1053 | 1.2243 | **1.1875** | 0.0663 | |  |
|  | **D55A** | C 9 | DFFNDFEDLFDDDAIQ | 1.6027 | 1.2358 | 0.8853 | 1.0270 | 1.0560 | 1.1842 | 1.4726 | 1.0634 | 1.0587 | 1.2851 | **1.1871** | 0.0730 | |  |
|  | **I56A** | C10 | DFFNDFEDLFDDDDAQ | 1.6138 | 1.2641 | 0.8800 | 0.9591 | 1.0276 | 1.2616 | 1.6616 | 1.1526 | 1.0733 | 1.2573 | **1.2151** | 0.0862 | |  |
|  | **Q57A** | C11 | DFFNDFEDLFDDDDIA | 1.5414 | 1.3641 | 0.9278 | 1.0285 | 1.0340 | 1.3898 | 1.5710 | 1.1879 | 1.0087 | 1.3004 | **1.2354** | 0.0772 | |  |

|  |  |  |  | **intensities as fold of C-CSNAP (A1)** | | | | | | | | | |  | |  | |
| --- | --- | --- | --- | --- | --- | --- | --- | --- | --- | --- | --- | --- | --- | --- | --- | --- | --- |
|  |  |  |  | Experiment #1 | | Experiment #2 | | Experiment #3 | | Experiment #4 | | Experiment #5 | |  |  | |  |
|  | **Residues** | **Position** | **sequence** | **left** | **right** | **left** | **right** | **left** | **right** | **left** | **right** | **left** | **right** | **Average fold change** | **standard error** | |  |
| **Phe to Trp substitution** | **F43W** | C12 | DWFNDFEDLFDDDDIQ | 1.5534 | 1.3821 | 0.9767 | 1.1693 | 1.1427 | 1.5293 | 1.7238 | 1.1283 | 0.9441 | 1.3603 | **1.2910** | 0.0867 | |  |
|  | **F44W** | C13 | DFWNDFEDLFDDDDIQ | 1.4732 | 1.3954 | 1.1153 | 1.0764 | 1.0769 | 1.4738 | 1.8258 | 1.1750 | 1.0053 | 1.2662 | **1.2883** | 0.0848 | |  |
|  | **F47W** | C14 | DFFNDWEDLFDDDDIQ | 1.1842 | 1.1679 | 0.9261 | 0.8987 | 0.9775 | 1.2511 | 1.6291 | 0.9710 | 0.9297 | 1.2689 | **1.1204** | 0.0762 | |  |
|  | **F51W** | C15 | DFFNDFEDLWDDDDIQ | 1.0829 | 1.0326 | 0.8765 | 0.7579 | 0.9049 | 1.1301 | 1.6444 | 0.7914 | 0.8400 | 1.2410 | **1.0302** | 0.0890 | |  |
|  | **F43,44W** | C16 | DWWNDFEDLFDDDDIQ | 1.4596 | 1.3608 | 1.2161 | 0.9851 | 1.0642 | 1.4681 | 2.3237 | 1.1090 | 0.9019 | 1.3448 | **1.3233** | 0.1342 | |  |
|  | **F43,47W** | C17 | DWFNDWEDLFDDDDIQ | 1.5889 | 1.5708 | 1.4424 | 1.1611 | 1.2333 | 1.7331 | 2.6056 | 1.4805 | 1.0216 | 1.4100 | **1.5247** | 0.1453 | |  |
|  | **F43,51W** | C18 | DWFNDFEDLWDDDDIQ | 1.2077 | 1.1175 | 1.2021 | 0.9687 | 0.9889 | 1.5562 | 2.2877 | 1.2083 | 0.7849 | 1.1631 | **1.2485** | 0.1390 | |  |
|  | **F44,47,51W** | C23 | DWFNDWEDLWDDDDIQ | 1.0818 | 1.1914 | 1.0070 | 1.0229 | 1.0518 | 1.8310 | 2.1664 | 1.2833 | 0.5518 | 0.9382 | **1.2126** | 0.2121 | |  |
|  | **F43,47,51W** | C24 | DWWNDWEDLTDDDDIQ | 0.5407 | 0.5259 | 0.5200 | 0.4965 | 0.4992 | 1.0466 | 1.3236 | 1.0978 | 0.2619 | 0.5419 | **0.6854** | 0.1801 | |  |
|  | **F43,44,47,51W** | D 2 | DWWNDWEDLWDDDDIQ | 1.1560 | 0.9155 | 0.8120 | 0.8690 | 0.9778 | 1.2404 | 0.9022 | 0.8643 | 0.6626 | 0.7687 | **0.9168** | 0.1653 | |  |

|  |  |  |  | **intensities as fold of C-CSNAP (A1)** | | | | | | | | | |  | |  | |
| --- | --- | --- | --- | --- | --- | --- | --- | --- | --- | --- | --- | --- | --- | --- | --- | --- | --- |
|  |  |  |  | Experiment #1 | | Experiment #2 | | Experiment #3 | | Experiment #4 | | Experiment #5 | |  |  | |  |
|  | **Residues** | **Position** | **sequence** | **left** | **right** | **left** | **right** | **left** | **right** | **left** | **right** | **left** | **right** | **Average fold change** | **standard error** | |  |
| **Asp to Glu substitution** | **D42E** | D 3 | EFFNDFEDLFDDDDIQ | 1.2985 | 1.1144 | 0.8090 | 0.8006 | 0.9587 | 1.1920 | 0.8253 | 0.8963 | 1.2065 | 0.9930 | **1.0094** | 0.0609 | |  |
|  | **D46E** | D 4 | DFFNEFEDLFDDDDIQ | 1.2336 | 1.1839 | 0.8044 | 0.9216 | 1.0487 | 1.1978 | 0.9142 | 0.9347 | 1.2050 | 1.0126 | **1.0457** | 0.0506 | |  |
|  | **D49E** | D 5 | DFFNDFEELFDDDDIQ | 1.1808 | 0.9146 | 0.6627 | 0.7444 | 0.9113 | 1.0463 | 0.9273 | 0.7705 | 1.0904 | 0.9868 | **0.9235** | 0.0541 | |  |
|  | **D52E** | D 6 | DFFNDFEDLFEDDDIQ | 1.3101 | 1.2556 | 0.8002 | 1.1488 | 1.1151 | 1.2622 | 1.2786 | 0.9646 | 1.2404 | 1.2058 | **1.1581** | 0.0538 | |  |
|  | **D53E** | D 7 | DFFNDFEDLFDEDDIQ | 1.3907 | 1.2258 | 0.7696 | 1.0251 | 1.0841 | 1.4114 | 1.2406 | 0.9582 | 1.1736 | 1.2212 | **1.1500** | 0.0655 | |  |
|  | **D54E** | D 8 | DFFNDFEDLFDDEDIQ | 1.3988 | 1.2663 | 0.7115 | 1.0067 | 1.0026 | 1.2341 | 1.2577 | 0.9918 | 1.0853 | 1.2827 | **1.1238** | 0.0675 | |  |
|  | **D55E** | D 9 | DFFNDFEDLFDDDEIQ | 1.3693 | 1.1572 | 0.7177 | 0.8554 | 0.9507 | 1.1673 | 1.1958 | 0.9914 | 1.1074 | 1.1841 | **1.0696** | 0.0636 | |  |
|  | **D42,46E** | D10 | EFFNEFEDLFDDDDIQ | 1.2677 | 1.1196 | 0.7261 | 0.8063 | 0.9152 | 1.1533 | 1.3276 | 0.9423 | 1.0772 | 1.2865 | **1.0622** | 0.0693 | |  |
|  | **D42,49E** | D11 | EFFNDFEELFDDDDIQ | 1.2263 | 1.1935 | 0.6949 | 0.8366 | 0.9229 | 1.1782 | 1.3497 | 0.9349 | 1.1356 | 1.3000 | **1.0773** | 0.0721 | |  |
|  | **D42,52E** | D12 | EFFNEFEDLFEDDDIQ | 1.0165 | 0.9343 | 0.7089 | 0.7692 | 0.9700 | 1.1628 | 1.4417 | 0.8681 | 1.0221 | 1.2535 | **1.0147** | 0.0741 | |  |
|  | **D42,53E** | D13 | EFFNDFEDLFDEDDIQ | 1.1578 | 1.1415 | 0.8400 | 0.7871 | 0.9025 | 1.2198 | 1.8177 | 1.0733 | 1.0443 | 1.3615 | **1.1346** | 0.0993 | |  |
|  | **D42,54E** | D14 | EFFNDFEDLFDDEDIQ | 1.0686 | 1.1142 | 0.7694 | 0.7894 | 0.8602 | 1.1678 | 1.4558 | 0.9192 | 0.8677 | 1.2979 | **1.0310** | 0.0765 | |  |
|  | **D42,55E** | D15 | EFFNDFEDLFDDDEIQ | 1.0647 | 1.0708 | 0.7472 | 0.6496 | 0.8116 | 1.1096 | 1.4848 | 1.0065 | 0.8714 | 1.2817 | **1.0098** | 0.0841 | |  |
|  | **D46,49E** | D16 | DFFNEFEELFDDDDIQ | 1.3397 | 1.3775 | 1.0691 | 0.9339 | 0.9371 | 1.3668 | 1.7587 | 1.1427 | 1.0756 | 1.6129 | **1.2614** | 0.0929 | |  |
|  | **D46,52E** | D17 | DFFNEFEDLFEDDDIQ | 1.2601 | 1.1744 | 0.9193 | 0.7850 | 0.8340 | 1.2425 | 1.7266 | 1.0630 | 1.0102 | 1.4263 | **1.1441** | 0.0958 | |  |
|  | **D46,53E** | D18 | DFFNEFEDLFDEDDIQ | 1.2036 | 0.9475 | 0.8828 | 0.6985 | 0.8297 | 1.3352 | 1.7452 | 0.9044 | 0.7496 | 1.2360 | **1.0532** | 0.1080 | |  |
|  | **D46,54E** | D19 | DFFNEFEDLFDDEDIQ | 1.1864 | 1.0957 | 0.9693 | 0.7109 | 0.7857 | 1.3254 | 2.0106 | 0.9793 | 0.8224 | 1.2414 | **1.1127** | 0.1250 | |  |
|  | **D46,55E** | D20 | DFFNEFEDLFDDDEIQ | 1.4459 | 1.4818 | 1.1784 | 0.9953 | 0.9089 | 1.5050 | 2.1861 | 1.2759 | 0.9049 | 1.6986 | **1.3581** | 0.1326 | |  |
|  | **D49,52E** | D21 | DFFNDFEELFEDDDIQ | 1.2701 | 1.3623 | 1.2490 | 1.0054 | 0.9028 | 1.6349 | 2.0031 | 1.2454 | 0.9943 | 1.9589 | **1.3626** | 0.1291 | |  |
|  | **D49,53E** | D22 | DFFNDFEELFDEDDIQ | 1.3211 | 1.4040 | 1.1180 | 0.9613 | 0.9989 | 1.5947 | 1.9606 | 1.0494 | 0.9503 | 1.8935 | **1.3252** | 0.1269 | |  |
|  | **D49,54E** | D23 | DFFNDFEELFDDEDIQ | 1.1601 | 1.1634 | 0.8244 | 0.7276 | 0.9437 | 1.2197 | 1.5970 | 0.9501 | 0.7969 | 1.5401 | **1.0923** | 0.1003 | |  |
|  | **D49,55E** | D24 | DFFNDFEELFDDDEIQ | 1.2542 | 1.4194 | 0.9839 | 0.7574 | 1.1287 | 1.4347 | 1.7639 | 0.9052 | 0.8193 | 1.7082 | **1.2175** | 0.1196 | |  |
|  | **D52,53E** | E 1 | DFFNDFEDLFEEDDIQ | 1.4743 | 1.1702 | 1.0146 | 1.0185 | 0.9795 | 1.3627 | 0.8141 | 1.0528 | 1.2430 | 1.1148 | **1.1244** | 0.0649 | |  |
|  | **D52,54E** | E 2 | DFFNDFEDLFEDEDIQ | 1.3670 | 1.0448 | 0.8546 | 0.8051 | 0.9045 | 1.2089 | 0.6844 | 0.9213 | 1.1956 | 0.9687 | **0.9955** | 0.0697 | |  |
|  | **D52,55E** | E 3 | DFFNDFEDLFEDDEIQ | 1.1393 | 0.8776 | 0.5939 | 0.5748 | 0.8010 | 0.9363 | 0.6220 | 0.7649 | 1.0403 | 0.7872 | **0.8137** | 0.0630 | |  |
|  | **D42,46,49E** | E 4 | EFFNEFEELFDDDDIQ | 1.1391 | 0.8576 | 0.5541 | 0.5733 | 0.7956 | 0.9401 | 0.6652 | 0.7322 | 1.0457 | 0.8035 | **0.8106** | 0.0640 | |  |
|  | **D42,46,52E** | E 5 | EFFNEFEDLFEDDDIQ | 1.2852 | 1.2019 | 0.7033 | 0.8244 | 0.9863 | 1.1994 | 0.9867 | 0.8748 | 1.2864 | 1.1164 | **1.0465** | 0.0677 | |  |
|  | **D42,46,53E** | E 6 | EFFNEFEDLFDEDDIQ | 1.2978 | 1.0555 | 0.6586 | 0.7908 | 0.8949 | 1.1478 | 1.0497 | 0.6571 | 1.1274 | 0.9774 | **0.9657** | 0.0711 | |  |
|  | **D42,46,54E** | E 7 | EFFNEFEDLFDDEDIQ | 1.4496 | 1.3250 | 0.7330 | 0.9388 | 1.0199 | 1.3332 | 1.2104 | 1.0375 | 1.2303 | 1.2021 | **1.1480** | 0.0716 | |  |
|  | **D42,46,55E** | E 8 | EFFNEFEDLFDDDEIQ | 1.1474 | 1.0270 | 0.5239 | 0.6997 | 0.7607 | 1.1027 | 1.0092 | 0.8244 | 1.0280 | 1.1036 | **0.9227** | 0.0694 | |  |
|  | **D42,49,52E** | E 9 | EFFNDFEELFEDDDIQ | 1.0877 | 1.0804 | 0.6603 | 0.7425 | 0.8450 | 1.1601 | 1.1508 | 0.8347 | 1.2269 | 1.3232 | **1.0112** | 0.0746 | |  |
|  | **D42,49,53E** | E10 | EFFNDFEELFDEDDIQ | 1.1819 | 1.3072 | 0.7601 | 0.8597 | 0.9454 | 1.3374 | 1.3641 | 0.9948 | 1.2944 | 1.5337 | **1.1579** | 0.0844 | |  |
|  | **D42,49,54E** | E11 | EFFNDFEELFDDEDIQ | 0.9278 | 0.8841 | 0.5860 | 0.6106 | 0.8117 | 1.1100 | 1.0262 | 0.7366 | 1.0571 | 1.1818 | **0.8932** | 0.0686 | |  |
|  | **D42,49,55E** | E12 | EFFNDFEELFDDDEIQ | 1.0184 | 0.9038 | 0.7097 | 0.6519 | 0.8127 | 1.1588 | 1.2842 | 0.7538 | 1.0700 | 1.3475 | **0.9711** | 0.0812 | |  |
|  | **D46,49,52E** | E13 | DFFNEFEELFEDDDIQ | 1.1552 | 1.2428 | 0.8701 | 0.9260 | 0.9661 | 1.4097 | 1.6951 | 1.0739 | 1.3265 | 1.7605 | **1.2426** | 0.1030 | |  |

| continued |  |  | **intensities as fold of C-CSNAP (A1)** | | | | | | | | | |  |  |
| --- | --- | --- | --- | --- | --- | --- | --- | --- | --- | --- | --- | --- | --- | --- |
|  |  |  | Experiment #1 | | Experiment #2 | | Experiment #3 | | Experiment #4 | | Experiment #5 | |  |  |
| **Residues** | **Position** | **sequence** | **left** | **right** | **left** | **right** | **left** | **right** | **left** | **right** | **left** | **right** | **Average fold change** | **standard error** |
| **D46,49,53E** | E14 | DFFNEFEELFDEDDIQ | 1.0069 | 1.0506 | 0.6696 | 0.6969 | 0.7442 | 1.1844 | 1.3249 | 0.8066 | 1.0159 | 1.4204 | **0.9920** | 0.0877 |
| **D46,49,54E** | E15 | DFFNEFEELFDDEDIQ | 0.9408 | 0.9766 | 0.5720 | 0.5824 | 0.6017 | 0.9830 | 1.1730 | 0.7676 | 0.8312 | 1.3187 | **0.8747** | 0.0844 |
| **D46,49,55E** | E16 | DFFNEFEELFDDDEIQ | 1.0317 | 1.0321 | 0.6712 | 0.6151 | 0.6274 | 1.0643 | 1.4109 | 0.8297 | 0.8896 | 1.3731 | **0.9545** | 0.0949 |
| **D49,52,53E** | E17 | DFFNDFEELFEEDDIQ | 1.2094 | 1.3336 | 0.9595 | 0.9059 | 0.7942 | 1.4435 | 1.7274 | 1.1474 | 1.1235 | 1.7287 | **1.2373** | 0.1076 |
| **D49,52,54E** | E18 | DFFNDFEELFEDEDIQ | 1.0763 | 0.9551 | 0.7483 | 0.7094 | 0.6858 | 1.1530 | 1.3104 | 0.7689 | 0.9009 | 1.3727 | **0.9681** | 0.0836 |
| **D49,52,55E** | E19 | DFFNDFEELFEDDEIQ | 1.1569 | 1.0050 | 0.9134 | 0.7702 | 0.7471 | 1.3599 | 1.6575 | 0.8615 | 0.9942 | 1.4438 | **1.0909** | 0.1021 |
| **D52,53,54E** | E20 | DFFNDFEDLFEEEDIQ | 1.2404 | 1.0658 | 0.8867 | 0.7559 | 0.8115 | 1.3555 | 1.5944 | 0.8653 | 0.9242 | 1.5459 | **1.1046** | 0.1032 |
| **D52,53,55E** | E21 | DFFNDFEDLFEEDEIQ | 1.4248 | 1.3911 | 1.0782 | 0.9150 | 0.9816 | 1.5588 | 2.0114 | 1.0911 | 1.0022 | 1.8126 | **1.3267** | 0.1259 |
| **D52,53,56E** | E22 | DFFNDFEDLFEDEEIQ | 1.2154 | 1.1697 | 0.9363 | 0.8257 | 0.9108 | 1.4025 | 1.7183 | 1.0158 | 0.8490 | 1.6777 | **1.1721** | 0.1101 |
| **D42,46,49,52E** | E23 | EFFNEFEELFEDDDIQ | 0.9809 | 0.9496 | 0.8449 | 0.7185 | 0.8989 | 1.2043 | 1.4176 | 0.7947 | 0.8436 | 1.6649 | **1.0318** | 0.1013 |
| **D42,46,49,53E** | E24 | EFFNEFEELFDEDDIQ | 1.2454 | 1.3845 | 0.8992 | 0.8018 | 1.0368 | 1.4192 | 1.6486 | 0.9608 | 0.8687 | 2.0006 | **1.2266** | 0.1297 |
| **D42,46,49,54E** | F 1 | EFFNEFEELFDDEDIQ | 1.3539 | 1.1678 | 0.9370 | 0.7534 | 0.9520 | 1.1742 | 0.7244 | 0.8890 | 1.4068 | 1.1069 | **1.0465** | 0.0781 |
| **D42,46,49,55E** | F 2 | EFFNEFEELFDDDEIQ | 1.0565 | 0.7393 | 0.7770 | 0.5904 | 0.8680 | 1.0774 | 0.5996 | 0.6664 | 1.0436 | 0.8392 | **0.8257** | 0.0616 |
| **D42,49,52,53E** | F 3 | EFFNDFEELFEEDDIQ | 1.0096 | 0.8061 | 0.6954 | 0.5788 | 0.8475 | 1.0099 | 0.6085 | 0.7216 | 1.1793 | 0.8269 | **0.8283** | 0.0638 |
| **D42,49,52,54E** | F 4 | EFFNDFEELFEDEDIQ | 1.2806 | 1.2401 | 0.8615 | 0.9192 | 1.1066 | 1.3577 | 0.9332 | 0.9431 | 1.3802 | 0.9273 | **1.0950** | 0.0675 |
| **D42,49,52,55E** | F 5 | EFFNDFEELFEDDEIQ | 1.0479 | 0.9605 | 0.6466 | 0.6747 | 0.8386 | 1.1400 | 0.8373 | 0.7660 | 1.2129 | 0.9054 | **0.9030** | 0.0630 |
| **D42,52,53,54E** | F 6 | EFFNDFEDLFEEEDIQ | 1.2011 | 1.3704 | 0.7819 | 0.8560 | 1.0176 | 1.4439 | 1.0790 | 0.9079 | 1.3567 | 1.0965 | **1.1111** | 0.0763 |
| **D42,52,53,55E** | F 7 | EFFNDFEDLFEEDEIQ | 1.1858 | 1.2521 | 0.6853 | 0.9102 | 0.9192 | 1.3458 | 1.1017 | 0.9656 | 1.1850 | 1.1096 | **1.0660** | 0.0653 |
| **D46,49,52,53E** | F 8 | DFFNEFEELFEEDDIQ | 0.8457 | 0.6958 | 0.4604 | 0.6054 | 0.7512 | 1.0741 | 0.9432 | 0.6514 | 0.9754 | 1.0099 | **0.8013** | 0.0668 |
| **D46,49,52,54E** | F 9 | DFFNEFEELFEDEDIQ | 0.9573 | 0.9441 | 0.5819 | 0.5913 | 0.7961 | 1.1763 | 1.0664 | 0.7032 | 1.1649 | 1.2720 | **0.9254** | 0.0830 |
| **D46,49,52,55E** | F10 | DFFNEFEELFEDDEIQ | 1.0767 | 1.3241 | 0.6469 | 0.6639 | 0.9295 | 1.4595 | 1.2511 | 0.9288 | 1.3137 | 1.5822 | **1.1176** | 0.1072 |
| **D49,52,53,54E** | F11 | DFFNDFEELFEEEDIQ | 0.8405 | 1.0271 | 0.6435 | 0.6864 | 0.7914 | 1.3015 | 1.1787 | 0.8836 | 1.1806 | 1.3104 | **0.9844** | 0.0830 |
| **D49,52,53,55E** | F12 | DFFNDFEELFEEDEIQ | 1.0504 | 1.3061 | 0.8019 | 0.8797 | 0.8768 | 1.4723 | 1.5496 | 0.9963 | 1.2664 | 1.5497 | **1.1749** | 0.0967 |
| **D52,53,54,55E** | F13 | DFFNDFEDLFEEEEIQ | 1.2924 | 1.4068 | 0.9184 | 0.9695 | 0.9472 | 1.6406 | 1.7333 | 1.1622 | 1.3240 | 1.6252 | **1.3020** | 0.1006 |
| **D42,46,49,52,53E** | F14 | EFFNEFEELFEEDDIQ | 1.2327 | 1.3339 | 0.8217 | 0.9664 | 0.8614 | 1.5490 | 1.6420 | 0.9822 | 1.1567 | 1.7860 | **1.2332** | 0.1128 |
| **D42,46,49,52,54E** | F15 | EFFNEFEELFEDEDIQ | 1.0071 | 0.9533 | 0.6223 | 0.6447 | 0.6508 | 1.1122 | 1.3401 | 0.7181 | 0.9806 | 1.5339 | **0.9563** | 0.1035 |
| **D42,46,49,52,55E** | F16 | EFFNEFEELFEDDEIQ | 1.1147 | 0.8613 | 0.7708 | 0.6644 | 0.6873 | 1.2361 | 1.6001 | 0.7063 | 0.9519 | 1.3000 | **0.9893** | 0.1047 |
| **D46,49,52,53,54E** | F17 | DFFNEFEELFEEEDIQ | 1.4429 | 1.4602 | 1.0530 | 0.9879 | 0.8653 | 1.5839 | 2.1153 | 1.1937 | 1.2735 | 1.7693 | **1.3745** | 0.1274 |
| **D46,49,52,54,55E** | F18 | DFFNEFEELFEDEEIQ | 1.2714 | 1.3996 | 0.9139 | 0.9139 | 0.7948 | 1.4656 | 1.5784 | 1.0372 | 1.0987 | 1.8276 | **1.2301** | 0.1116 |
| **D46,49,52,53,55E** | F19 | DFFNEFEELFEEDEIQ | 1.2086 | 1.1391 | 0.9369 | 0.9030 | 0.7731 | 1.5375 | 1.6534 | 0.8885 | 1.0830 | 1.4767 | **1.1600** | 0.1015 |
| **D46,49,53,54,55E** | F20 | DFFNEFEELFDEEEIQ | 1.3623 | 1.5019 | 1.1927 | 1.1561 | 1.0841 | 1.8400 | 2.2763 | 1.1206 | 1.1980 | 1.8982 | **1.4630** | 0.1362 |
| **D49,52,53,54,55E** | F21 | DFFNDFEELFEEEEIQ | 1.2552 | 1.0717 | 0.8610 | 0.7800 | 0.8504 | 1.5206 | 1.8116 | 0.8739 | 1.0177 | 1.7535 | **1.1796** | 0.1292 |
| **D42,46,49,52,53,55E** | F22 | EFFNEFEELFEEEDIQ | 1.2507 | 1.3400 | 0.9276 | 0.9125 | 1.0634 | 1.5898 | 2.0496 | 1.0009 | 0.9351 | 1.7140 | **1.2784** | 0.1307 |
| **D42,46,49,53,54,55E** | F23 | EFFNEFEELFDEEEIQ | 0.9469 | 1.0066 | 0.7866 | 0.7272 | 0.9979 | 1.2998 | 1.3413 | 0.7717 | 0.7482 | 1.6954 | **1.0322** | 0.1065 |
| **D42,46,49,52,54,55E** | F24 | EFFNEFEELFEDEEIQ | 0.9428 | 1.2297 | 0.8184 | 0.8311 | 1.1381 | 1.4027 | 1.4631 | 0.8229 | 0.7971 | 1.7109 | **1.1157** | 0.1087 |
| **D42,46,49,52,53, 54E** | G 1 | EFFNEFEELFEEEDIQ | 1.1648 | 1.0575 | 0.7793 | 0.5456 | 0.8906 | 1.1335 | 0.5629 | 0.7600 | 1.1869 | 1.2035 | **0.9285** | 0.0853 |
| **D46,49,52,53,54,55E** | G 2 | DFFNEFEELFEEEEIQ | 1.1981 | 1.1382 | 0.9021 | 0.5723 | 1.0237 | 1.2612 | 0.7058 | 0.8240 | 1.3873 | 1.1204 | **1.0133** | 0.0861 |
| **D42,49,52,53,54,55E** | G 3 | EFFNDFEELFEEEEIQ | 1.1174 | 0.9755 | 0.8222 | 0.6019 | 0.9166 | 1.1632 | 0.6539 | 0.8061 | 1.2449 | 0.9053 | **0.9207** | 0.0704 |
| **D42,46,49,52,53,54,56E** | G 4 | EFFNEFEELFEEEEIQ | 1.2685 | 1.3185 | 0.8670 | 0.8491 | 1.0270 | 1.4403 | 0.9396 | 1.0223 | 1.4389 | 0.9891 | **1.1160** | 0.0762 |

|  |  |  |  | **intensities as fold of C-CSNAP (A1)** | | | | | | | | | |  | |  | |
| --- | --- | --- | --- | --- | --- | --- | --- | --- | --- | --- | --- | --- | --- | --- | --- | --- | --- |
|  |  |  |  | Experiment #1 | | Experiment #2 | | Experiment #3 | | Experiment #4 | | Experiment #5 | |  |  | |  |
|  | **Residues** | **Position** | **sequence** | **left** | **right** | **left** | **right** | **left** | **right** | **left** | **right** | **left** | **right** | **Average fold change** | **standard error** | |  |
| **d-AA substitution** | **D42d** | G 5 | dFFNDFEDLFDDDDIQ | 1.3993 | 1.4598 | 0.7856 | 0.8316 | 1.0609 | 1.4125 | 1.0498 | 0.9789 | 1.3252 | 0.8787 | **1.1182** | 0.0863 | |  |
|  | **F43f** | G 6 | DfFNDFEDLFDDDDIQ | 1.0934 | 1.1515 | 0.5904 | 0.6826 | 0.8878 | 1.2262 | 0.9020 | 0.8156 | 1.0727 | 0.8158 | **0.9238** | 0.0693 | |  |
|  | **F44f** | G 7 | DFfNDFEDLFDDDDIQ | 0.9950 | 1.1962 | 0.5009 | 0.6506 | 0.7698 | 1.1411 | 0.8688 | 0.7313 | 1.0468 | 0.9317 | **0.8832** | 0.0739 | |  |
|  | **N45n** | G 8 | DFFnDFEDLFDDDDIQ | 0.9480 | 1.1546 | 0.4759 | 0.6269 | 0.7487 | 1.1575 | 0.9459 | 0.7887 | 1.0087 | 0.9702 | **0.8825** | 0.0736 | |  |
|  | **D4d6** | G 9 | DFFNdFEDLFDDDDIQ | 1.1504 | 1.4467 | 0.6541 | 0.8543 | 0.8740 | 1.4723 | 1.3835 | 0.9879 | 1.2038 | 1.3371 | **1.1364** | 0.0942 | |  |
|  | **F47f** | G10 | DFFNDfEDLFDDDDIQ | 1.1255 | 1.1331 | 0.5675 | 0.7107 | 0.7834 | 1.3091 | 1.3433 | 0.8053 | 1.1808 | 1.2735 | **1.0232** | 0.0933 | |  |
|  | **E48e** | G11 | DFFNDeDLFDDDDIQ | 1.2038 | 1.4034 | 0.7825 | 0.9467 | 0.8979 | 1.5260 | 1.5198 | 1.0311 | 1.3607 | 1.4547 | **1.2126** | 0.0928 | |  |
|  | **D49d** | G12 | DFFNDFEdLFDDDDIQ | 1.1437 | 1.1876 | 0.5694 | 0.6906 | 0.7476 | 1.2342 | 1.3468 | 0.8614 | 1.1323 | 1.2883 | **1.0202** | 0.0924 | |  |
|  | **L50l** | G13 | DFFNDFEDlFDDDDIQ | 1.3043 | 1.4122 | 0.6882 | 0.7879 | 0.8102 | 1.4853 | 1.5515 | 0.9875 | 1.2601 | 1.5272 | **1.1814** | 0.1109 | |  |
|  | **F51f** | G14 | DFFNDFEDLfDDDDIQ | 1.0633 | 1.2809 | 0.5218 | 0.6988 | 0.6383 | 1.1747 | 1.3220 | 0.9176 | 0.9941 | 1.3376 | **0.9949** | 0.0986 | |  |
|  | **D52d** | G15 | DFFNDFEDLFdDDDIQ | 1.1025 | 1.1706 | 0.5481 | 0.6228 | 0.6439 | 1.1532 | 1.3998 | 0.7958 | 1.0026 | 1.3280 | **0.9767** | 0.1018 | |  |
|  | **D53d** | G16 | DFFNDFEDLFDdDDIQ | 1.3989 | 1.3723 | 0.6843 | 0.7266 | 0.7396 | 1.3195 | 1.7957 | 0.8322 | 1.0212 | 1.3196 | **1.1210** | 0.1247 | |  |
|  | **D54d** | G17 | DFFNDFEDLFDDdDIQ | 1.5352 | 1.6425 | 0.8976 | 0.9012 | 0.9147 | 1.6416 | 2.1213 | 1.0343 | 1.1066 | 1.5251 | **1.3320** | 0.1397 | |  |
|  | **D55d** | G18 | DFFNDFEDLFDDDdIQ | 1.4363 | 1.4841 | 0.8795 | 0.8572 | 0.9498 | 1.7207 | 1.8974 | 0.9587 | 1.0178 | 1.5649 | **1.2767** | 0.1288 | |  |
|  | **I56i** | G19 | DFFNDFEDLFDDDDiQ | 1.7132 | 1.8564 | 1.2232 | 1.1482 | 1.1480 | 2.0434 | 2.4264 | 1.2993 | 1.1058 | 1.6742 | **1.5638** | 0.1505 | |  |
|  | **Q57q** | G20 | DFFNDFEDLFDDDDIq | 1.5102 | 1.8800 | 1.1616 | 1.0906 | 1.1986 | 2.0081 | 2.1652 | 1.3448 | 1.1238 | 1.7797 | **1.5262** | 0.1340 | |  |

|  |  |  |  | **intensities as fold of C-CSNAP (A1)** | | | | | | | | | |  | |  | |
| --- | --- | --- | --- | --- | --- | --- | --- | --- | --- | --- | --- | --- | --- | --- | --- | --- | --- |
|  |  |  |  | Experiment #1 | | Experiment #2 | | Experiment #3 | | Experiment #4 | | Experiment #5 | |  |  | |  |
|  | **Residues** | **Position** | **sequence** | **left** | **right** | **left** | **right** | **left** | **right** | **left** | **right** | **left** | **right** | **Average fold change** | **standard error** | |  |
| **Non-proteogenic AA substitution** | **N-Met-D** | H13 | N-Me-Asp-FFNDFEDLFDDDDIQ | 1.4566 | 1.7962 | 0.7152 | 0.9895 | 0.8836 | 1.6535 | 1.7197 | 1.0118 | 1.3663 | 1.4459 | **1.3038** | 0.1263 | |  |
|  | **3x(LL-Aib)** | H14 | LL-Aib-LL-Aib-LL-Aib-GGG-DFFNDFEDLFDDDDIQ | 1.2814 | 1.9277 | 0.7073 | 1.1291 | 0.8476 | 1.8424 | 1.7380 | 1.2336 | 1.1737 | 1.6317 | **1.3512** | 0.1392 | |  |
|  | **+Aib(46,52,58)** | H15 | DFFN-Aib-FEDLF-Aib-DDDIQ-Aib | 0.4043 | 0.7210 | 0.1949 | 0.3301 | 0.3326 | 0.7753 | 0.6092 | 0.4361 | 0.5928 | 0.8025 | **0.5199** | 0.0699 | |  |
|  | **+Aib(45,50,56)** | H16 | DFF-Aib-DFED-Aib-FDDDI-Aib-Q | 0.6927 | 0.9720 | 0.4832 | 0.6026 | 0.6526 | 1.2044 | 1.5394 | 0.6821 | 0.7092 | 0.9444 | **0.8483** | 0.1073 | |  |
|  | **+Aib(46,52,57)** | H17 | DFFN-Aib-FEDLF-Aib-DDDI-Aib | 0.7679 | 0.8542 | 0.3535 | 0.4019 | 0.5013 | 1.0337 | 1.0367 | 0.5126 | 0.7846 | 0.8445 | **0.7091** | 0.0834 | |  |
|  | **+Aib(54,58)** | H18 | DFFNDFEDLFDD-Aib-DIQ-Aib | 1.0429 | 0.8897 | 0.6916 | 0.6906 | 0.9950 | 1.4159 | 1.6829 | 0.6718 | 0.9779 | 1.1087 | **1.0167** | 0.1089 | |  |
|  | **+Aib(42,48,54)** | H19 | Aib-FFNDF-Aib-DLFDD-Aib-DIQ | 1.3253 | 1.7109 | 0.9783 | 1.1237 | 1.2089 | 1.9920 | 2.2180 | 0.9097 | 1.2198 | 1.7272 | **1.4414** | 0.1480 | |  |
