# Supplementary material for: The C-terminal tail of CSNAP attenuates the CSN complex
Source: Life Sci Alliance. 2023 Jul 17;6(10):e202201634. doi: 10.26508/lsa.202201634 (PMC10355216; doi:10.26508/lsa.202201634)
Supplement: Supplementary file 3 [file LSA-2022-01634_TableS3.docx]

**Table S3. Primers used to design constructs in phyg-Cerulean used in Figure 2 and S2.**

| Insert | Forward Primer | Reverse Primer |
| --- | --- | --- |
| -^1^M^-41^A (C-CSNAP) | GACTTTTTTAACGATTTTGAAGATC | CATGCTAGCGGATCTGAC |
| -^42^D | TTTTTTAACGATTTTGAAGATCTTTTTG | CATGCTAGCGGATCTGAC |
| -^42^DF | TTTAACGATTTTGAAGATCTTTTTGATG | CATGCTAGCGGATCTGAC |
| -^57^Q | ACCGGTGGAGGAGGAAGC | GATGTCATCATCATCAAAAAGATCTTCAAAATCG |
| -^56^IQ | ACCGGTGGAGGAGGAAGC | GTCATCATCATCAAAAAGATCTTCAAAATCGTTAAAAAAG |
| -^55^DIQ | ACCGGTGGAGGAGGAAGC | ATCATCATCAAAAAGATCTTCAAAATCGTTAAAAAAGTC |
| -^54^DDIQ | ACCGGTGGAGGAGGAAGC | ATCATCAAAAAGATCTTCAAAATCGTTAAAAAAGTCC |
